# Effects of 8 weeks of moderate physical training on body composition, lipid profile, inflammatory markers, and physical activity in middle aged females

**DOI:** 10.3389/fendo.2025.1734772

**Published:** 2026-01-09

**Authors:** Maha Sellami, Shamma Almuraikhy, Najeha Anwardeen, Parveen B. Nizamuddin, Haitam Othman, Noora Alathba, Noor Alareer, Mohamed A. Elrayess

**Affiliations:** 1 Sport Coaching Department, College of Sport Sciences, Qatar University, Doha, Qatar; 2 Biomedical Research Center, Qatar University (QU) Health, Qatar University, Doha, Qatar; 3 Department of Biomedical Science, College of Health Science, Qatar University (QU) Health, Qatar University, Doha, Qatar; 4 College of Medicine, Qatar University (QU) Health, Qatar University, Doha, Qatar

**Keywords:** inflammatory cytokines, middle-aged females, moderate physical activity, oxidative stress, telomere length

## Abstract

**Introduction:**

Moderate physical training is widely recommended for reducing cardiovascular risk in adults. However, its effects on body composition, lipid metabolism, inflammatory markers, and physical activity levels, along with the potential use of cytokines as biomarkers for evaluating training effectiveness, in middle-aged and older adults (30–60 years) remain to be investigated.

**Methods:**

Participants aged 30–60 years underwent an 8-week of moderate-intensity aerobic training (MAT) program. Pre- and post-intervention assessments included body mass index (BMI); fat mass, muscle mass. Oxidative stress markers (superoxide dismutase [SOD] and catalase). Inflammatory cytokines (IL-6, IL-8, IL-10, IL-22, TNF-α [Tumor Necrosis Factor-alpha], MCP-1[Monocyte Chemoattractant Protein-1], IL-1RA). Lipid profile [total cholesterol, LDL-C [Low-Density Lipoprotein Cholesterol], HDL-C [High-Density Lipoprotein Cholesterol]. Telomere length, metabolic equivalent of task [MET], and the duration of vigorous and moderate physical activity per week.

**Results:**

After 8 weeks of training, significant reductions were observed in BMI, fat free mass, muscle mass, and inflammatory cytokines (IL-22 &TNF-α). Oxidative stress markers showed increase in SOD level. Lipid profile analysis revealed decreases in total cholesterol, LDL-C, and HDL-C. Notably, there were significant increases in MET and moderate physical activity per week, indicating improved physical activity levels.

**Conclusions:**

The reduction in pro-inflammatory cytokines aligns with the established anti-inflammatory benefits of regular exercise, contributing to the lower risk of chronic disease. However, the simultaneous decrease in anti-inflammatory cytokines suggests a complex and potentially age-specific immune adaptation to moderate training. Further investigation is warranted to clarify the implications of these immune responses and to refine exercise recommendations for optimal metabolic and immune health in these middle-aged females.

## 1 Introduction

For requirements for a specific article type please refer to the Article Types on any Frontiers journal Manuscript Formatting Physical activity (PA) is widely recognized as a cornerstone of health promotion and disease prevention, particularly in middle-aged adults. Regular exercise is associated with favorable changes in body composition, lipid metabolism, and inflammatory status, all of which contribute to reduce risk of chronic diseases such as cardiovascular disease, type 2 diabetes, and metabolic syndrome (1, 2). However, the extent and direction of these adaptations can vary significantly depending on age, exercise intensity, duration, and individual baseline characteristics.

Four to eight weeks interventions yield sustained enhancements in IS, cardiovascular performance, and metabolic function, sometimes independent of notable changes in body composition. Recent studies, for example, show that eight weeks of aerobic training in young women leads to significant improvements in IS and cardiovascular capacity, while four weeks yield moderate but less pronounced effects (3).

Continued regular PA over 8 to 12 weeks leads to lasting adaptations, including improved body composition, reduced inflammation in adipose tissue, lowered ectopic lipid deposition, increased mitochondrial density, and boosted antioxidant status in skeletal muscle. These cumulative changes reinforce metabolic health, lower insulin resistance (IR), and protect against chronic disease (4, 5).

Evidence from recent guidelines, including the American College of Sports Medicine (ACSM) and US CDC(Centers for Disease Control and Prevention), confirms that PA of varying durations consistently promotes metabolic health, supports weight management, and reduces cardio-metabolic risk. Guidelines recommend adults engage in aerobic exercise and strength training at least twice weekly, with total weekly aerobic activity of at least 150min at moderate intensity or 75min at vigorous intensity (6). The effectiveness of PA is influenced by its type, volume, and structure. Moderate intensity exercise offers substantial cardiovascular and metabolic benefits, though excessive strenuous exercise may carry risks for certain individuals. Thus, thoughtful periodization and personalized exercise planning are critical for optimizing health outcomes (7, 8).

Previous research has demonstrated that exercise interventions typically lead to improvements in lipid profiles, including reductions in total cholesterol and low-density lipoprotein (LDL), as well as increases in high-density lipoprotein (HDL) in adults across various age groups (2). Moreover, exercise is known to modulate inflammatory pathways, with the anti-inflammatory IL-10 often increasing in response to PA, especially following intense or prolonged exercise sessions (9, 10).

Cytokine levels can serve as valuable biomarkers for monitoring muscle damage and inflammatory responses associated with physical exercise. Evaluating individual cytokine profiles enables the development of personalized training programs, facilitating optimized recovery and performance. Consequently, the use of cytokine biomarkers holds significant promise for refining exercise protocols and improving overall health outcomes (9).

In our previous study, we investigated the effects of eight weeks of moderate-intensity aerobic training (MAT) in young females aged 20–30 years. Our data revealed improved IS, metabolic flexibility, and anti-inflammatory cytokine profiles in young women (3). Building on these findings, the present study extends the investigation to older females aged 30–60 years to assess the influence of age on physiological and metabolic responses to MAT. Specifically, we evaluate the impact of MAT on body composition, insulin sensitivity, physical performance, lipid profile, telomere length, inflammatory markers, and oxidative stress parameters. This comparative approach aims to clarify whether age modulates the beneficial effects of moderate aerobic training across these critical health indicators. Understanding this relationship is essential for utilizing PA as a non-pharmacological strategy for health promotion and disease prevention (11).

## 2 Materials and methods

### 2.1 Study participants:

Forty non-obese, apparently healthy women aged 30–60 years participated in this study. Inclusion criteria included a BMI of 20–30 kg/m² and the absence of cardiovascular conditions, type 2 diabetes, muscle degeneration, blood clots, or neurological disorders. A small number of borderline BMI cases were included due to measurement rounding or recruitment timing. All participants provided written informed consent prior to participation. The study protocols were approved by Qatar University (QU-IRB 1798-EA/23) in compliance with the regulations of the Qatar Ministry of Public Health (MoPH).

### 2.2 Training session

Participants were engaged in an aerobic training session for 8 weeks, following the American College of Sports Medicine (ACSM) and American Heart association (AHA) recommendations (12–16). The exercise regimen comprised aerobic exercises with progressive intensity (40–60%) of HRmax (Maximum Heart Rate) and 50% of VO2 (Volume of Oxygen) peak initially, progressing to 60–70% by the 8th week. All participants trained three days/week for 50 minutes per session. The Metabolic Equivalent of Task (MET) values were adjusted based on the International Physical Activity Questionnaire (IPAQ) responses to quantify daily activities. MET was utilized for intensity and energy expenditure, expressed similarly for individuals of different weights. A total of 51 women were initially enrolled in the study. Forty participants (78%) completed the entire 8-week MAT intervention and all pre/post assessments, while 11 participants withdrew. Reasons for withdrawal included relocation (n = 2), lack of attendance starting from week 3 (n = 3), social/family factors (n = 5), and a minor musculoskeletal injury unrelated to the training program (n = 1). Adherence to the training sessions among completers was high, with a mean attendance of 91% (range: 85–100%). Compliance with exercise intensity (40–70% HRmax, progressively increased across the 8 weeks) was monitored via continuous heart rate manual measuring from the coaches.

### 2.3 Study measures

Body composition (TANITA, Tanita Corporation, Japan) was measured before and after training, early in the morning, after 8 h of fasting. TANITA will provide measurements for weight (kg), body fat percentage (%), fat mass (kg), fat-free mass (kg), muscle mass (kg), body mass index (BMI), and height (cm). Muscle mass is a part of fat-free mass, but fat-free mass includes many other tissues beyond muscle. This distinction is important when interpreting body composition results, as increases in fat-free mass can reflect gains in muscle, water, or other tissues, not muscle alone (17).

### 2.4 Clinical parameters and cytokines measurements

Fasting blood samples were collected before and after completing 8 weeks intervention. Fasting blood sugar, total cholesterol, triglycerides, HDL and LDL were measured using the clinical chemistry analyzer Mindray BS240 according to manufacturer’s instructions. Insulin levels were measured in serum samples using Insulin ELISA kit (Mercodia, UK) according to manufacturer’s instructions. Homeostatic Model Assessment of insulin resistance (HOMA-IR) was used to assesses IR using the formula: HOMA= Fasting blood glucose (mmol/L) × Fasting insulin (mIU/mL)/22.5. Body fat, fat free mass, fat mass, and muscle mass was measured using TANITA body composition monitor. Custom Premix Human Cyto Panel A 09 Plex (HCYTA-60K-08C, Millipore) was used to simultaneously profile cytokines, including IL-1RA, IL-6, IL-8/CXCL8, MCP-1/CCL2, TGF-α and TNF-α using Luminex™ FLEXMAP 3D, according to manufacturer’s instructions. Separate standard curves were used to validate the assay for the detection and quantification of cytokines according to manufacturer’s instructions using Xponent software. Activities of superoxide dismutase and catalase were determined using the colorimetric activity assays (EIACATC and EIASODC, respectively), according to manufacturer’s instructions (ThermoFisher Scientific, USA).

### 2.5 Measurement of telomere length

PureLink^®^ Genomic DNA Kits (Invitrogen, Life Technologies, Carlsbad, CA, USA) were used for the isolation of genomic DNA from the clotted blood at the bottom of the serum tubes, according to the manufacturer’s instructions as described previously (18). Telomere length was measured using Absolute Human Telomere Length Quantification qPCR Assay Kit (ScienCell, Carlsbad, CA, USA) according to the manufacturer’s instructions. Briefly, two qPCR reactions were prepared for each genomic DNA sample: one with telomere (TL) and one with single copy reference (SCR) primer stock solutions. qPCR reactions were prepared by adding a genomic DNA template (5 ng/µL) to the primer stock solution (TL or SCR) and GoldNStart TaqGreen qPCR master mix. qPCR was run using an initial denaturation of 95°C for 10 min, followed by 32 cycles of denaturation at 95°C for 20 s, annealing at 52°C for 20 s, and extension at 72°C for 45 s using StepOne™ Real-Time PCR System (ThermoFisher). For quantification of TL, ΔCq (TL) was quantified by assessing the TL cycle number difference between the two genomic DNA samples (sample of interest and the reference genomic DNA sample with known telomere length). For SCR, ΔCq (SCR) was assessed by quantifying the SCR cycle number difference between the two genomic DNA samples (sample of interest and the reference genomic DNA sample with known telomere length). ΔCq was calculated as ΔCq (TL) − ΔCq (SCR). Fold change was assessed as 2 −ΔΔCq, and the TL was expressed as a T/S ratio.

### 2.6 Statistical methods

Normality of the data distribution was assessed using the Shapiro–Wilk test. For variables that followed a normal distribution, a paired t-test was performed to compare before and after intervention values. For non-normally distributed data, the Wilcoxon signed-rank test was applied. A two-tailed p value < 0.05 was considered statistically significant. All analyses were conducted using GraphPad Prism (version 18; GraphPad Software, San Diego, CA, USA). Multiple testing correction was performed using Benjamini–Hochberg FDR for each set of parameters (cytokines, lipids, blood sugar, physical activity, and oxidative stress markers) to adjust for false positive error.

## 3 Results

### 3.1 Baseline characteristics of participants

**Table 1** provides baseline characteristics of the participants in this study, grouped into before- and after-training. The data show that after exercise intervention, participants experienced modest reductions in BMI, LDL, total cholesterol, inflammatory cytokines (IL-22 & TNF-α), and muscle/fat-free mass. Significant decreases in BMI (FDR = 0.049), LDL (FDR = 0.0087), total cholesterol (FDR = 0.0026), and several cytokines, though weight and fat mass remained largely unchanged. Antioxidant enzyme SOD significantly increased, while catalase changes were non-significant. Overall, results suggest improvements in lipid profile, inflammatory markers, and oxidative stress defense, but at the cost of reduced muscle mass.

**Table 1:** Baseline characteristics of participants.

Clinical Traits	Before	After	P-value	Median/Mean Δ	CI (95-97%)	FDR
Age	41 (37 – 47.75)		-	-	-	
BMI	31 (26.4 - 33.45)	29.8 (25.8 - 32.25)	0.0245	-0.30	-1.70 to 0.20	**0.0490**
FBS (mmol/L)	5.3 (5.15 - 5.75)	5.2 (4.95 - 5.85)	0.2102	-0.10	-0.30 to 0.00	0.2102
Insulin (mU/L)	9.26 (5.82 - 14.72)	7.47 (5.31 - 14.61)	0.201	-0.77	-2.10 to 0.83	0.2102
HOMA-IR	2.27 (1.34 - 3.68)	1.69 (1.08 - 4.27)	0.076	-0.28	-0.57 to 0.08	0.2012
Catalase (U/mL)	20.43 (17.6 - 25.86)	22.96 (17.85 - 27.01)	0.211	1.43	-1.14 to 2.62	0.2110
SOD (U/mL)	0.71 (0.63 - 0.73)	0.77 (0.68 - 0.89)	0.0004	0.06	0.01 to 0.17	**0.0008**
IL-1β (pg/ml)	39.68 (11.7 - 84.09)	38.78 (7.04 - 73.42)	0.2629	-4.58	-17.81 to 2.62	0.3380
IL-1RA (pg/ml)	25.52 (18.8 - 51.11)	21.78 (13.6 - 42.73)	0.0286	-4.06	-9.03 to -1.05	0.0622
IL-6 (pg/ml)	7.15 (5.02 - 17.54)	7.26 (3.85 - 13.26)	0.0595	-1.12	-3.46 to 0.32	0.0893
IL-8 (pg/ml)	19.78 (13.9 - 24.88)	19.02 (11.76 - 28.84)	0.8525	-1.37	-2.88 to 2.15	0.8525
IL-10 (pg/ml)	15.84 (11.52 - 26.97)	13.15 (8.78 - 17.44)	0.0303	-2.31	-5.93 to -1.12	0.0622
IL-22 (pg/ml)	751 (407.9 - 1291)	612.5 (324.7 - 1160)	0.0107	-137.40	-189.00 to -18.89	**0.0481**
MCP-1 (pg/ml)	681.6 (302.6)	601.4 (262.8)	0.0346	-80.21	-154.30 to -6.11	0.0622
TGF-α (pg/ml)	24.61 (11.6 - 55.25)	22.48 (14.16 - 44.66)	0.3678	-1.30	-7.21 to 2.57	0.4128
TNF-α (pg/ml)	79.61 (53.14 - 113.2)	64.07 (39.59 - 94.56)	0.0002	-16.28	-20.58 to -3.21	**0.0018**
TC (mmol/L)	4.65 (3.93 - 5.18)	4.05 (3.44 - 4.75)	0.0013	-0.43	-0.60 to -0.11	**0.0026**
TG (mmol/L)	0.86 (0.63 - 1.36)	0.80 (0.62 - 1.37)	0.7421	-0.07	-0.18 to 0.06	0.7421
HDL (mmol/L)	1.33 (1.1 - 1.56)	1.12 (0.94 - 1.39)	0.0001	-0.14	-0.25 to -0.04	**0.0004**
LDL (mmol/L)	2.85 (2.58 - 3.52)	2.68 (2.2 - 3.23)	0.0065	-0.26	-0.38 to -0.03	**0.0087**
Walk (min/week)	594 (247.5-1386)	594 (231-1151)	0.6335	-147	-396 to 264	0.6335
Vigorous (min/week)	0 (0-1260)	0 (0-960)	0.5135	0	-320 to 0	0.6335
Total MET value/week	1386 (498-2585)	2267 (735-3765)	0.0233	471	-105 to 1741	**0.0466**
Moderate (min/week)	320 (0-720)	600 (280-1320)	0.021	240	0 to 420	**0.0466**
Fat-free mass (Kg)	45.6 (40.85 - 50.75)	42.5 (39.4 - 46.3)	0.0031	-1.20	-3.94 to 0.00	**0.0186**
Muscle mass (Kg)	39.3 (35.3 - 44.3)	29.9 (22.7 - 39.9)	0.0119	-2.60	-15.10 to 0.10	**0.0357**
Weight (Kg)	74.9 (67.18 - 85.9)	73.8 (66.38 - 79.55)	0.1773	-0.80	-2.50 to 0.50	0.2659
Body fat %	40.1 (36.3 - 42.2)	40.4 (36.9 - 47.8)	0.4298	-0.20	-1.40 to 3.20	0.5157
Fat mass (Kg)	30.15 (24.38 - 37.7)	30.6 (25.93 - 35.73)	0.7417	-0.95	-1.70 to 1.00	0.7417

Data are presented as median (interquartile range)/mean (SD). P-values were calculated using the Wilcoxon matched-pairs signed rank test or paired t-test according to Shapiro Wilk’s normality test. The median change (Δ) and its 95% confidence interval (CI) reflect the differences post-intervention. Multiple testing correction using FDR was performed for each set of parameters.

The bold values indicate an FDR < 0.05.

### 3.2 Effect of training on body composition parameters

**Figure 1:**
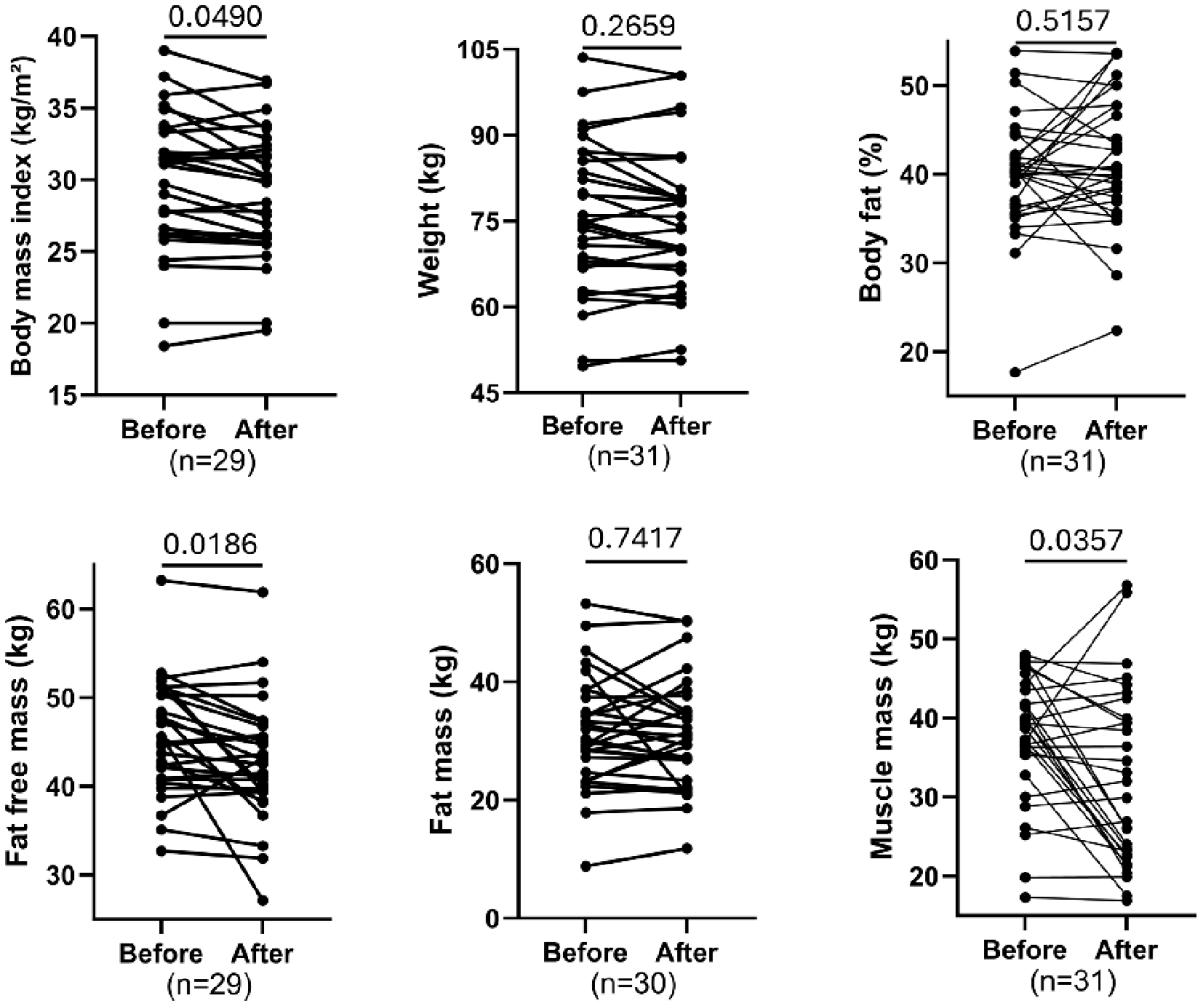
Comparing paired body measurements before and after exercise. Each plot displays individual participant trajectories. P-values were calculated using the Wilcoxon matched-pairs signed rank test or paired t-test according to Shapiro Wilk’s normality test. FDR<0.05 is significant.

**Figure 1** presents paired “before and after exercise intervention” plots for several body composition parameters. There was a statistically significant reduction in body mass index (BMI; FDR=0.049) and fat-free mass (FDR = 0.0186), as well as muscle mass (FDR = 0.0357) following the intervention. In contrast, there were no significant changes in weight (FDR = 0.2659), body fat percentage (FDR = 0.5157), or fat mass (FDR = 0.7417). The significant reductions in BMI and lean mass parameters suggest changes in body composition were driven by losses in fat-free and muscle mass rather than reductions in fat mass or overall body weight.

### 3.3 Effect of training on blood sugar

**Figure 2:**
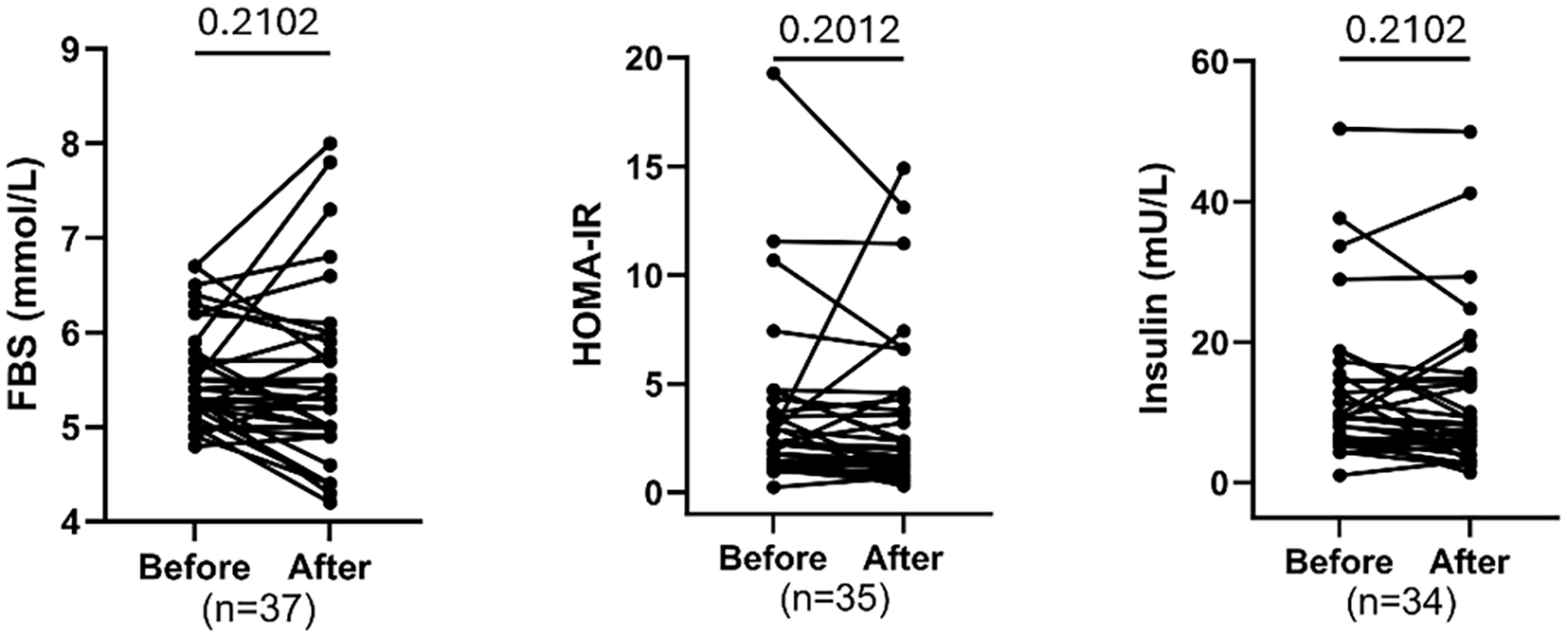
Comparing fasting glucose, insulin levels, and insulin resistance (HOMA-IR) before and after exercise. Each plot displays individual participant trajectories. P-values were calculated using the Wilcoxon matched-pairs signed rank test or paired t-test according to Shapiro Wilk’s normality test. FDR<0.05 is significant.

The paired comparison of blood sugar before and after intervention showed that 8 weeks of MAT exhibited no significant reduction in FBS, insulin and HOMA-IR after training, although a trend of reduced HOMA-IR was noted (FDR = 0.2012) (**Figure 2**).

### 3.4 Effects of training on lipid profile parameters

**Figure 3:**
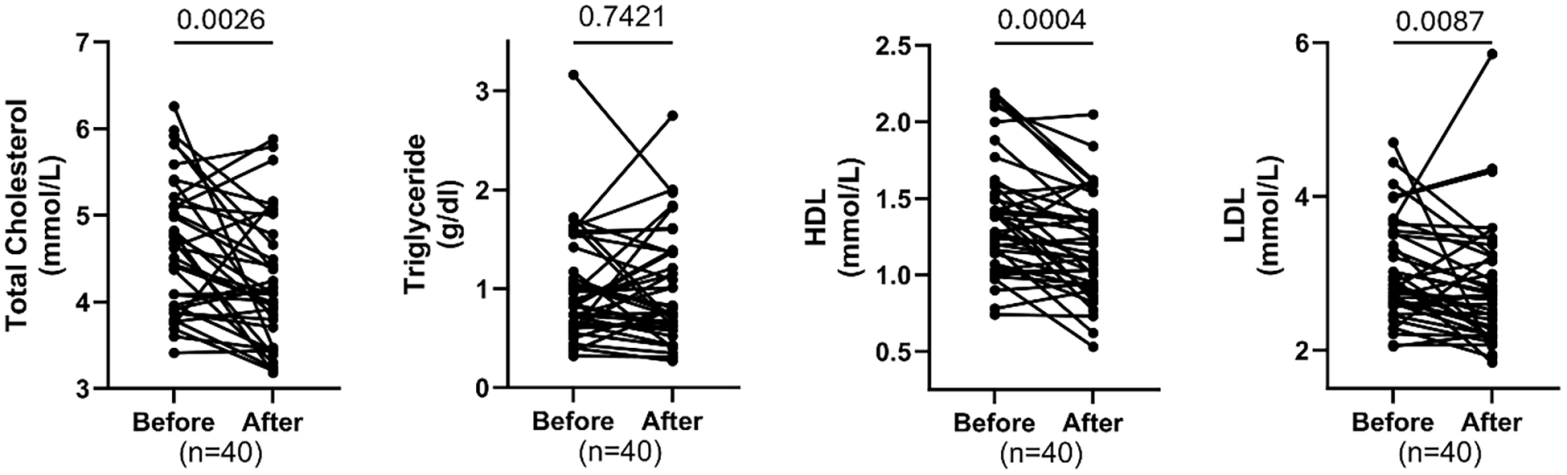
Comparing total cholesterol, triglyceride, HDL & LDL cholesterol before and after exercise. Each plot displays individual participant trajectories. P-values were calculated using the Wilcoxon matched-pairs signed rank test or paired t-test according to Shapiro Wilk’s normality test. FDR <0.05 is significant.

**Figure 3** shows significant reductions in total cholesterol (FDR=0.0026) and LDL cholesterol (FDR = 0.0087), and a significant decrease in HDL cholesterol (FDR = 0.0004) following the intervention. Triglyceride levels did not change significantly (FDR = 0.7421). Individual trajectories indicate consistent decreases for total and LDL cholesterol, while HDL also declined for most participants. Overall, the intervention improved total and LDL cholesterol but unexpectedly reduced HDL, with no impact on triglycerides.

### 3.5 Changes in physical activity levels and energy expenditure

**Figure 4:**
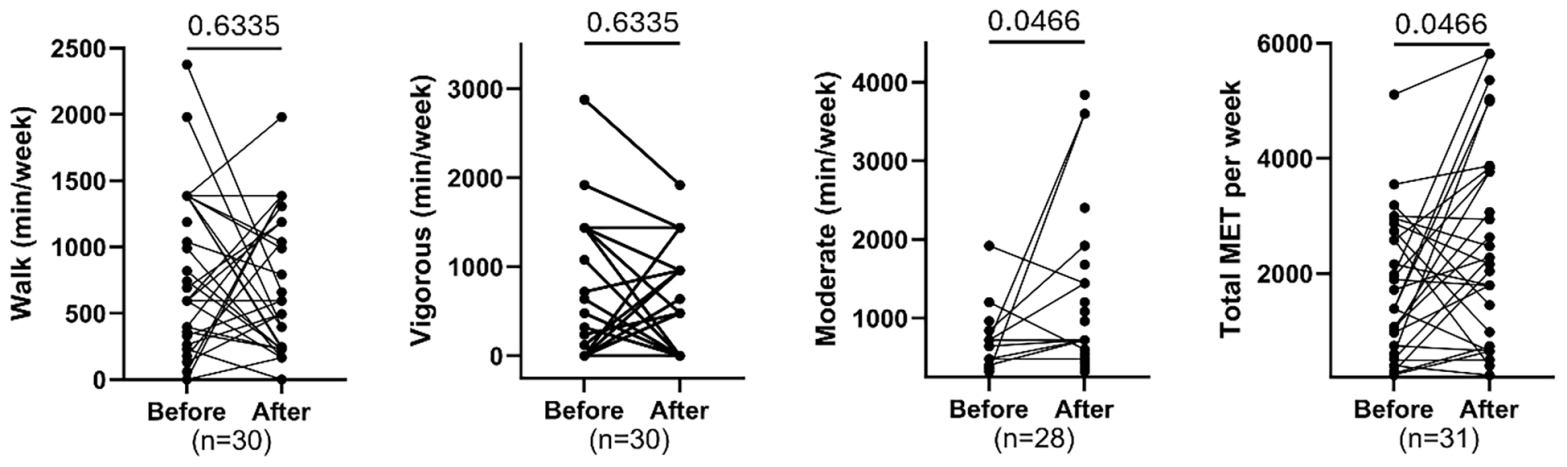
Comparing paired total walking (min/week), vigorous, moderate physical activity and total MET before and after exercise. Each plot displays individual participant trajectories. P-values were calculated using the Wilcoxon matched-pairs signed rank test or paired t-test according to Shapiro Wilk’s normality test. FDR <0.05 is significant.

**Figure 4** illustrates changes in physical activity following the intervention. Walking (FDR = 0.6335) and vigorous activity (FDR = 0.6335) showed no significant change, but there was a significant increase in time spent on moderate-intensity activities (FDR = 0.0466) and in total weekly MET value (FDR = 0.0466). This suggests that, while overall walking and vigorous activity remained unchanged, participants increased their moderate physical activity and overall energy expenditure after the intervention.

### 3.6 Changes in cytokine and chemokine levels before and after training

**Figure 5:**
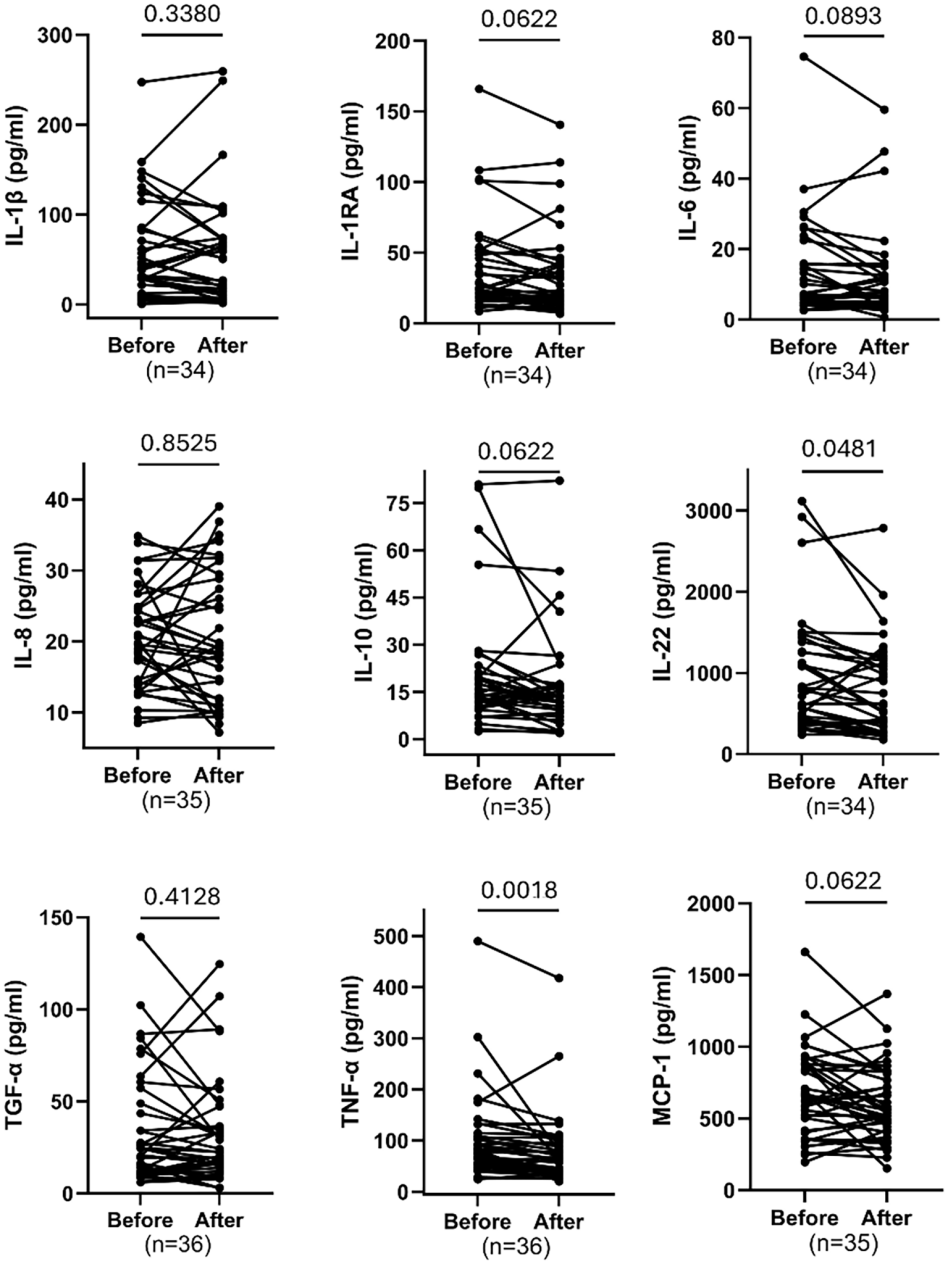
Comparing paired cytokines including IL-1β, IL-1RA, IL-6, IL-8, IL-10, IL-22, MCP-1, TNF-α and TGF-α before and after exercise. Each plot displays individual participant trajectories. P-values were calculated using the Wilcoxon matched-pairs signed rank test or paired t-test according to Shapiro Wilk’s normality test. FDR <0.05 is significant.

**Figure 5** displays paired comparisons of multiple cytokine and chemokine concentrations (in pg/ml) measured before and after an intervention. Statistically significant reductions were observed in IL-22 (FDR = 0.0481) and TNF-α(FDR = 0.0018). No significant changes were detected for IL-1β (FDR = 0.3380),IL-6(FDR = 0.0893), IL-8FDR=0.8525), TGF-α(FDR = 0.4128), IL-1RA (FDR = 0.0622), IL-10 (FDR = 0.0622) &MCP-1 (FDR = 0.0622). This suggests the intervention selectively decreased specific inflammatory mediators.

### 3.7 Changes in catalase and SOD levels before and after training

**Figure 6:**
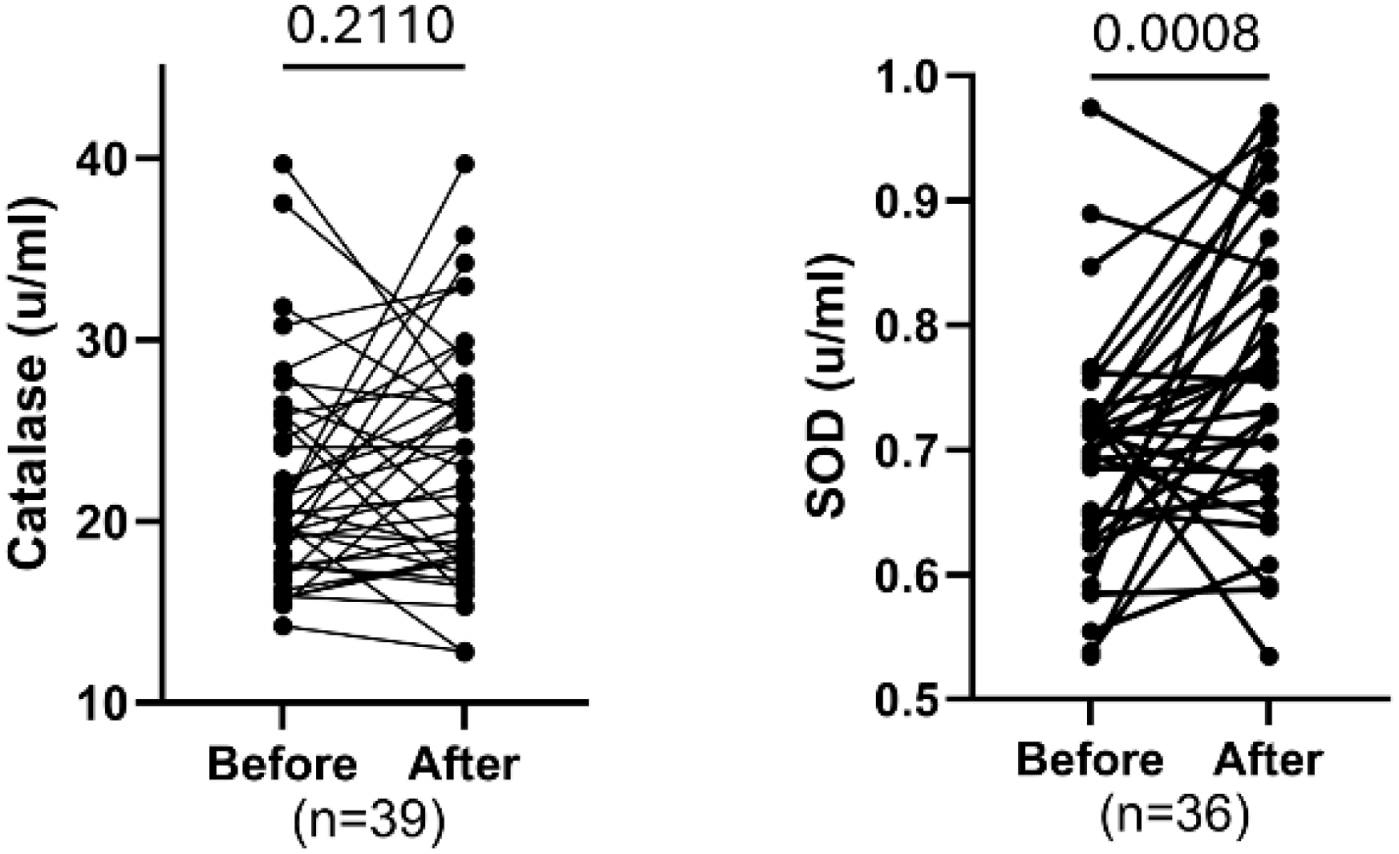
Comparing paired oxidative stress markers catalase and SOD before and after exercise. Each plot displays individual participant trajectories. P-values were calculated using the Wilcoxon matched-pairs signed rank test or paired t-test according to Shapiro Wilk’s normality test. FDR <0.05 is significant.

The intervention did not result in a significant change in catalase activity, as levels before and after were similar (FDR=0.2110). In contrast, SOD activity increased significantly following the intervention (FDR = 0.0008), indicating that exercise particularly boosted defenses against superoxide radicals, but not necessarily against hydrogen peroxide, highlighting a selective antioxidant adaptation in adults (**Figure 6**).

### 3.8 Changes in telomere length before and after training

**Figure 7:**
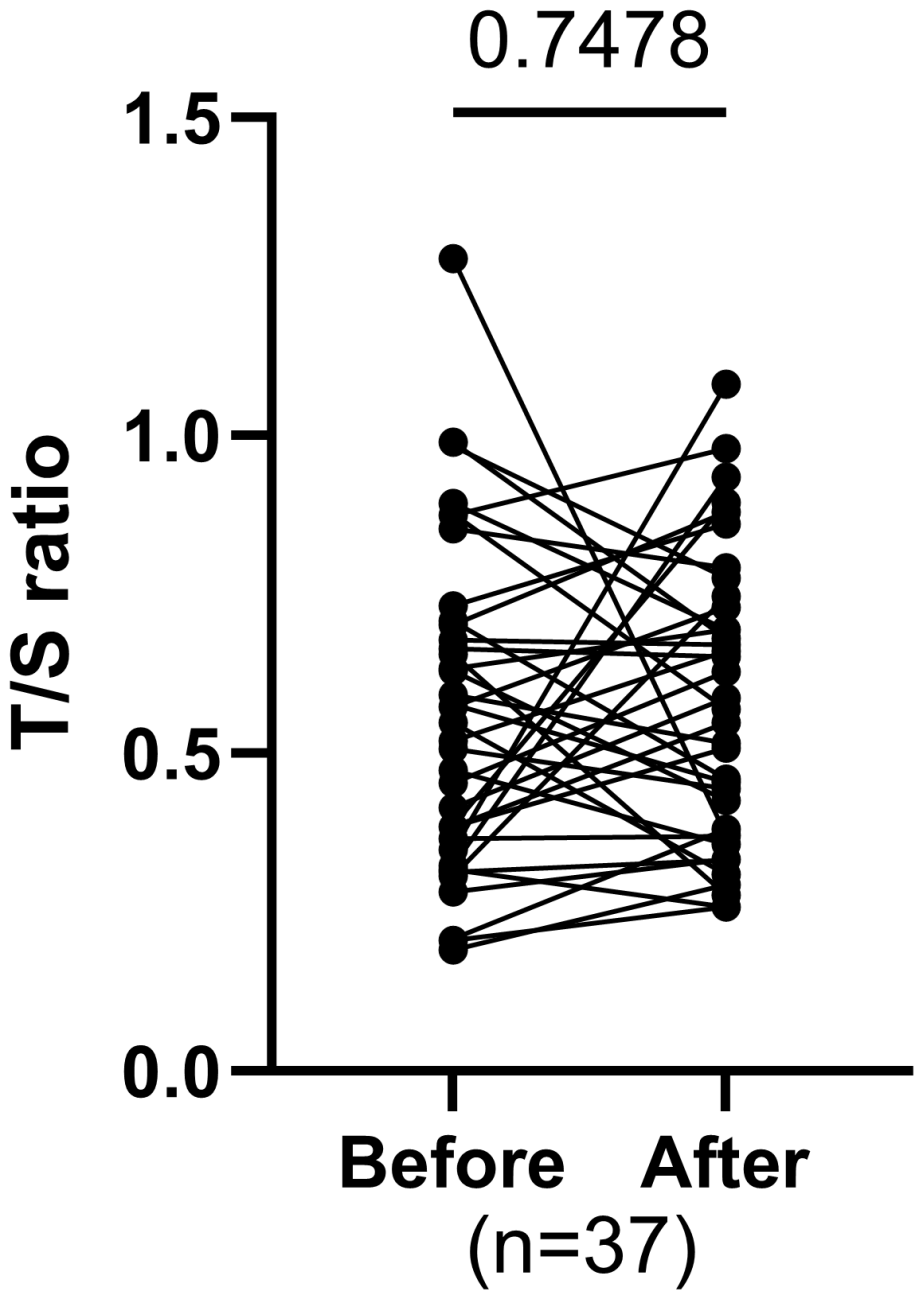
Comparing paired T/S ratio before and after exercise. P-values were calculated using the paired t-test. P value<0.05 is significant.

The T/S ratio, measured before and after the intervention in individual subjects, shows no statistically significant difference (p=0.7478), **Figure 7**).

## 4 Discussion

A growing body of evidence shows that engaging in moderate physical training produces substantial health benefits for middle-aged adults, including improvements in body composition, lipid profiles, antioxidant defenses, and inflammatory status. In this study, we investigated the impact of 8 weeks of aerobic training on lean to overweight women aged 30–60 years. The primary aim was to determine the optimal duration of exercise training needed to induce physiological adaptations, with a particular focus on glucose metabolism markers, lipid profiles, antioxidant defenses, inflammatory markers, and telomere length (TL), in order to explore the metabolic pathways associated with each training duration. Acute bouts of exercise rapidly enhance insulin signaling and glucose uptake in skeletal muscle, outcomes detectable within hours post-activity and lasting up to 24–48 hours. Even a single session of moderate aerobic exercise can trigger temporary improvements in insulin sensitivity (IS) and activate crucial metabolic pathways, such as GLUT4 translocation and AMPK signaling (4).

Our emerging data showed significant reduction in cytokines levels including IL-22and TNF-α, associated with 8 weeks MAT in women aged 30–60 years. Although IL-1RA, IL-10, and MCP-1 showed nominal significance based on unadjusted p-values, these effects were not maintained after FDR correction. This indicates that the findings may be exploratory rather than conclusive. As such, we have interpreted these cytokine changes as possible trends that require confirmation in larger, adequately powered studies.

Reduction in circulating IL-22 levels aligns with emerging evidence that moderate exercise can modulate immune-mediated cytokines and is particularly relevant to chronic, low-grade inflammation in middle-aged women. IL-22 plays roles in tissue repair, barrier integrity, and inflammation regulation and its decrease may represent an adaptive response improving tissue homeostasis in metabolic dysregulation (19, 20). Responses vary with training type, intensity, and participant health (21); for example, 16 weeks of moderate-intensity continuous training reduced IL-22 by 17%, while HIIT (High-Intensity Interval Training) showed no change, and combined aerobic–resistance training produced similar decreases with accompanying metabolic benefits. Lower IL-22 may act as a biomarker of improved metabolic health rather than the direct cause, as seen in T2DM and Polycystic Ovary Syndrome (PCOS) where moderate exercise reduces inflammation and improves insulin sensitivity (17, 18). Experimental studies indicate that IL-22 has regulatory effects on metabolic homeostasis, influencing glucose metabolism and adipose tissue health (22, 23).

Additionally, our results associated with a reduction in TNF-α, consistent with evidence that moderate physical training lowers this key inflammatory cytokine both acutely and chronically (24, 25). Even single bouts of moderate-intensity exercise can transiently decrease TNF-α, while ongoing training, such as 4 week or 12-week aerobic or combined programs produces sustained reductions, often independent of body composition changes (24, 26, 27). This anti-inflammatory effect is partly mediated by exercise-induced modulation of cytokine release, including β2-adrenergic receptor activation on monocytes (27–30), and helps reduce the risk of chronic disease associated with systemic inflammation (11, 25).

During exercise, contracting skeletal muscles release myokines like IL-6, which suppress TNF-α, promoting an anti-inflammatory environment and supporting long-term immune modulation (31). Collectively, these adaptations indicate that moderate physical training reduces levels of TNF-α and IL-22 through a multifaceted mechanism. This underscores the potential of moderate exercise as a powerful, non-pharmacological strategy to combat chronic inflammation and promote systemic health in women aged 30 to 60.

Further investigation is warranted to clarify the implications of these immune responses and to refine exercise recommendations for optimal metabolic and immune health in this population.

Eight weeks of physical training resulted in a significant increase in the oxidative stress marker, SOD with no change observed in catalase levels. SOD is a primary antioxidant enzyme, and its increased activity following exercise typically reflects adaptive enhancement of the body’s antioxidant defense system.

A recent meta-analysis revealed that exercise does not uniformly improve antioxidant enzymes, with no overall change in SOD, glutathione peroxidase, or catalase. SOD increases were seen mainly in individuals with BMI <25, those engaging in resistance or multicomponent training, and in 1–12-week programs. Effects varied by exercise type, intensity, age, and gender, with older adults showing reduced SOD responses and aerobic exercise being less effective than resistance, underscoring the need for personalized exercise plans to optimize antioxidant defenses (32–34).

Additional studies show that high-intensity interval training significantly increases SOD but not catalase, while low-to-moderate intensity exercise yields inconsistent effects. Physical fitness is generally linked to reduced oxidative stress, largely through upregulated SOD rather than catalase, which remains unchanged across most protocols. In elderly women, moderate aerobic training boosts SOD without affecting catalase, supporting exercise as a strategy to enhance antioxidant capacity with aging (35–37).

Consistent with a substantial body of research, eight weeks of structured exercise significantly reduced BMI, supporting evidence from systematic reviews that regular aerobic or combined training consistently improves body composition and metabolic health across adult age groups (38–41).

In contrast, our result associated with reduction in muscle mass & fat-free mass, which is atypical, as most evidence from resistance and mixed training interventions reports either maintenance or increases in muscle mass, particularly with adequate nutrition and appropriate training intensity (42–44). Possible reasons for this discrepancy include aspects of the training protocol, insufficient nutrition, age-related decreases in muscle-building capacity, or differences in measurement methods. The lack of data on diet, medication, and menopausal status further limits the interpretation of our results. Additionally, body composition was assessed by BIA (e.g., TANITA), which is sensitive to hydration status, recent food intake, and pre-test physical activity; BIA can over- or underestimate fat-free/muscle mass relative to DXA or MRI (23). Substantial individual-level error has been reported, with single readings sometimes deviating from reference methods by several kilograms (24). The absence of repeated/duplicate/averaged measurements increases the risk that outlier readings could skew results. For fat mass, BIA is generally more accurate than for muscle mass, but considerable error exists at both ends, and BIA estimates of muscle mass in particular can show substantial bias relative to DXA or MRI (45).

Muscle plays a critical role in maintaining health, function, and metabolic stability in midlife women. The observed loss of muscle mass during this stage has significant clinical implications, especially in the context of sarcopenia, the age-related loss of skeletal muscle mass and strength (46).

Additionally, our study demonstrated that MAT associated with significant reductions in total cholesterol, LDL, and HDL levels. Favorable reductions in total cholesterol and LDL were observed, consistent with the well-established cardio-protective benefits of exercise. Numerous recent meta-analyses and systematic reviews confirm that aerobic training, in particular, effectively lowers both total cholesterol and LDL, thereby improving cardiovascular risk profiles (47–51). These improvements are directly linked to reduced incidence of atherosclerosis and coronary heart disease.

Aerobic exercise improves overall lipid profiles, but its effect on HDL varies with factors like exercise type, intensity, duration, and individual characteristics, including age, race, body mass, baseline HDL levels, diet, and medication use (52). Interpretation of our results is further limited by the lack of data on diet, medication use, and menopausal status. Uncontrolled Diet, dietary composition and recent fat intake can cause rapid and notable shifts in HDL levels, independent of exercise. Without dietary monitoring, these changes could confound results (53). Lipid-lowering drugs, including statins, can decrease or blunt increases in HDL despite exercise, masking the expected effect (52). While regular physical activity often maintains or modestly increases HDL, several studies have reported either negligible changes or, slight reductions in HDL, especially during short-term or moderate-intensity interventions in middle-aged females. Most frequently, HDL cholesterol remains stable or improves, as demonstrated by meta-analyses and systematic reviews (38, 49, 54). However, Short-term HDL reductions may occur during rapid weight loss or dietary restriction, due to accelerated lipid mobilization and clearance, and can persist until weight stabilizes (38, 54). Additionally Short interventions (<8 weeks) or solely moderate-intensity training rarely increase HDL, especially in individuals with normal or high baseline levels (49, 50). Notably, aerobic exercise significantly improves HDL levels in middle-aged and older individuals, whereas resistance and stretching exercises appear to have no significant effect (55). Hormonal status, baseline metabolic risk, and dietary intake also influence HDL responses, particularly in women. For example, pre and post-menopausal status or caloric restriction can attenuate or reverse the expected rise in HDL, as shown in studies focused on middle-aged women (56). Importantly, even if HDL decreases slightly, moderate exercise still yields significant cardio metabolic benefits, particularly when LDL, total cholesterol, and inflammation are reduced. Such modest declines should not overshadow overall cardiovascular improvements (55).

Eight weeks of MAT associated with significantly increased MET values and weekly minutes of moderate physical activity, indicating greater exercise volume and intensity. A 2025 study found that young females who completed eight weeks of moderate aerobic training showed increases in MET and physical activity capacity (3). A 12-week exercise-based cardiac rehabilitation (CR) program led to significant increases in peak METs for women with normal and overweight BMI categories. Higher METs are closely associated with better cardiovascular and metabolic health (57, 58). Another study showed that moderate-to-intensive exercise in sedentary middle-aged women significantly increases METs, resulting in health improvements (59).

In our study, telomere length did not change after eight weeks of MAT, consistent with evidence that short-term, moderate-intensity exercise are typically insufficient to produces measurable telomere effects (60–62). Effect on telomere length is exploratory due to short intervention duration. Telomere changes occur gradually, as inflammation and oxidative stress reductions from exercise accumulate over time (60, 62, 63). Thus, although telomere length was unchanged, eight weeks of moderate exercise still conferred cardio-metabolic and anti-inflammatory benefits that may, over the long term, support telomere maintenance.

## 5 Conclusion

In conclusion, comparison of the effects of MAT between young women aged 20–30 years (3) (as presented in our previous study) and older women aged above 30 years (from our current study) reveals important age-related differences in physiological adaptations. In younger women, MAT primarily enhanced metabolic indicators such as improved HOMA-IR, reduced fasting insulin and triglyceride levels, and elevated MET, reflecting greater insulin sensitivity and cardiorespiratory fitness. In contrast, older women exhibited more pronounced benefits in lipid profiles (total cholesterol & LDL-C), while results showed reduction in HDL-C. In addition to that results showed reductions in inflammatory cytokines (IL-22 & TNF-α) and higher oxidative stress marker (SOD). MAT on older females showed reduction on BMI, muscle mass & Fat-free mass with no significant change on fat mass or body fat percentage. These findings suggest that while MAT confers health benefits across the lifespan, the specific physiological improvements may vary with age, with younger individuals experiencing greater gains in metabolic flexibility and older adults showing enhanced cardio-metabolic and anti-inflammatory responses. Cytokine responses to exercise are highly complex and influenced by multiple factors including baseline immune status, methodological differences in cytokine assessment, and the physiological heterogeneity inherent in aging populations. Given this complexity, there is a clear need for larger, controlled trials to elucidate the nuances of cytokine dynamics and to confirm age-related differences in adaptations to exercise. Finally, the absence of change in telomere length suggests that the duration of training plays a critical role in influencing telomere dynamics.

Future studies should assess the long-term persistence of age-specific benefits from moderate aerobic training and investigate underlying mechanisms, including hormonal influences. Personalized, age-tailored exercise potentially combined with nutrition and advanced biomarker profiling may optimize health outcomes for women across the lifespan. Studies should also account for baseline fitness, incorporate measures of immune variability.

## 6 Study limitations

We acknowledge several limitations in our study that warrant consideration and future investigation. These include primarily the small sample size and the absence of a control group. In addition, the lack of generalizability of our findings, as the cohort comprised non-obese women only, means findings may not extend to men, people with obesity, or those with chronic conditions. The lack of data on diet, medication, and menopausal status further limits the interpretation of our results. The short study duration further constrains interpretation. Telomere measurements, in particular, should be considered exploratory given the limited timeframe. The study may have been underpowered to detect changes in metabolic and cytokine endpoints. Internal inconsistency in muscle mass likely reflects methodological or measurement limitations. The absence of repeated, duplicate, or averaged measurements increases the risk that outlier readings could skew results. It also is important to note that for several outcomes, including BMI, fasting blood sugar, insulin, HOMA-IR, catalase, IL-1, IL-6, IL-8, TGF-β, muscle mass, fat-free mass, weight, body fat percentage, fat mass, and walking or vigorous physical activity time, the confidence intervals for the estimated effects cross zero. Accordingly, these results should be interpreted as suggestive but not definitive. In contrast, larger and more reliably estimated effects, such as the effects on SOD, IL-1RA, IL-10, IL-22, MCP-1, TNF-α, HDL, LDL, and total cholesterol, where the confidence intervals do not cross zero, provide stronger evidence for genuine intervention effects.

## Data Availability

The raw data supporting the conclusions of this article will be made available by the authors, without undue reservation.
