# Supplementary figures and images for: Effects of 8 weeks of moderate physical training on body composition, lipid profile, inflammatory markers, and physical activity in middle aged females
Source: Front Endocrinol (Lausanne). 2026 Jan 9;16:1734772. doi: 10.3389/fendo.2025.1734772 (PMC12827193; doi:10.3389/fendo.2025.1734772)

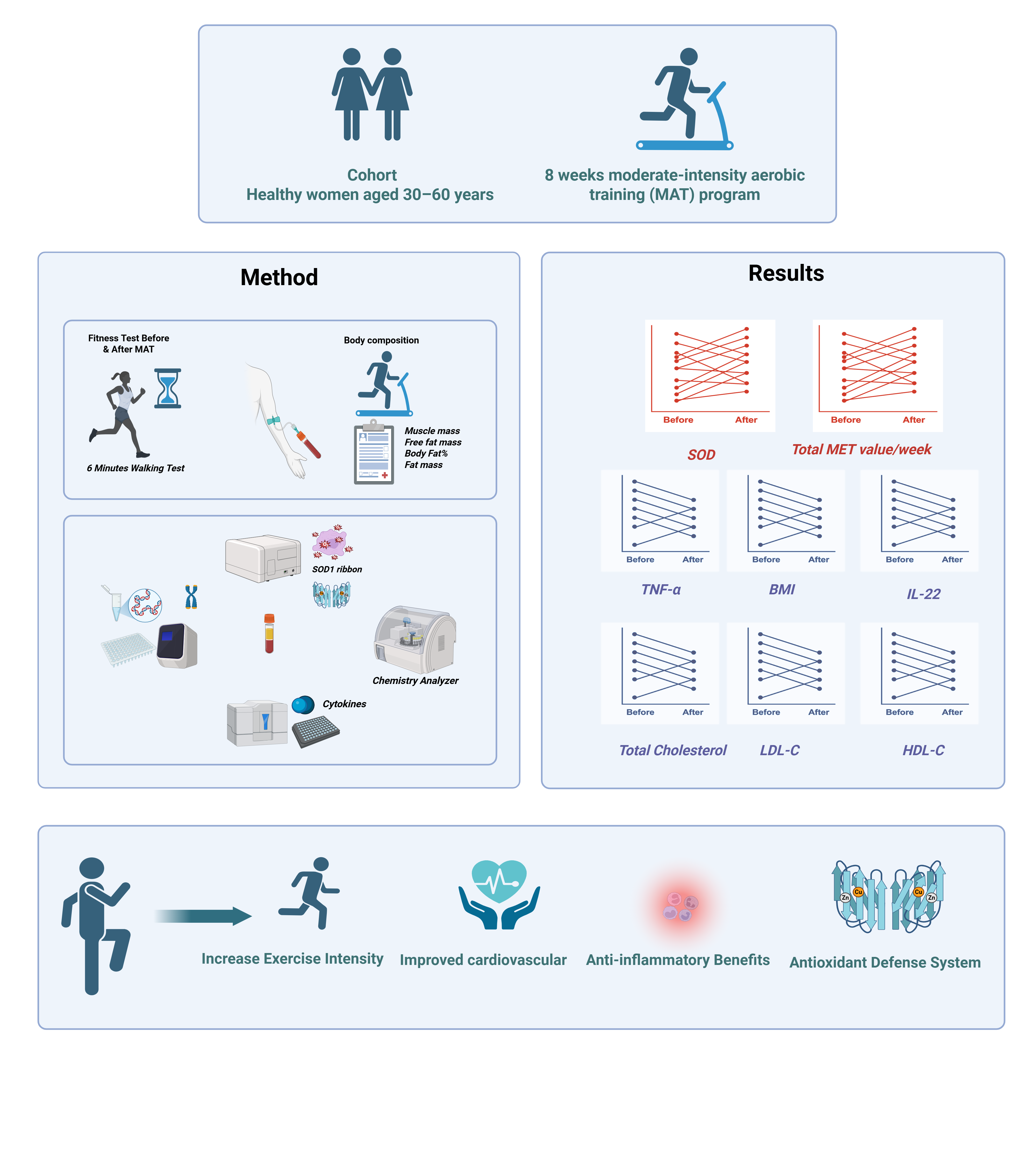

Supplement: Supplementary file 1 [file Image1.png]
